# Does socioeconomic status impact the relationship between school absence and outcomes?

**DOI:** 10.1007/s13384-022-00535-2

**Published:** 2022-05-17

**Authors:** Anna Mooney, Gerry Redmond, Billingsley Kaambwa

**Affiliations:** 1 Faculty of Medicine, Health and Human Sciences, Macquarie University, Sydney, Australia; 2 College of Government, Business and Law, Flinders University, Adelaide, Australia; 3 Health Economics, College of Medicine and Public Health, Flinders University, Adelaide, Australia

**Keywords:** Absence, Belonging, Outcomes, Socioeconomic, Survey data

## Abstract

Absence from school, especially frequent or prolonged absence, is acknowledged as a potential factor in school dropout and suboptimal academic achievement. The issue of absence from school took on added significance in 2020 with the onset of the COVID-19 crisis, which resulted in schooling interruptions in several jurisdictions. However, there is little agreement in the literature on the exact relationship between absence and school outcomes as a function of socioeconomic status (SES). Using nationally representative pre-COVID longitudinal data of young Australians aged 12–13 and 14–15, this paper examines the relationship between absence from school on the one hand and school belonging and academic achievement (numeracy and reading test scores) on the other. The paper also examines whether SES intersects this relationship. Controlling for gender, prior educational achievement, computer access, and time spent doing homework, the study finds that absence impacts belonging, but that SES does not significantly influence this relationship. The effect of absence on reading is not significant either. However, absence is associated with numeracy outcomes, with the strongest associations among low SES young people at age 14. Policy implications of these findings are discussed.

**Supplementary Information:**

The online version contains supplementary material available at 10.1007/s13384-022-00535-2.

## Introduction

The longer-term impact of school closures on educational outcomes of young people as a result of COVID-19 health restrictions is not yet known. In response to the COVID-19 pandemic, the Australian federal government commissioned rapid response reports from leading research institutes to assess the impacts of remote learning on educational outcomes for vulnerable young people (Australian Government, 2020). Among the synthesis of evidence on the matter, the papers advised that school closures and prolonged remote learning could potentially result in poorer educational outcomes for Australian primary and secondary school students (Centre for International Research on Education Systems, 2020; Rapid Research Information Forum, 2020). The groups of students identified by these reports and other research as being at particularly greater risk of poorer outcomes included those from a low socioeconomic status (SES), those whose first language is not English, those living in remote areas and those with special learning needs (Centre for International Research on Education Systems, 2020; Mupenzi et al., 2020; Rapid Research Information Forum, 2020).

The Centre for International Research on Education Systems (CIRES, 2020) found that school closures present additional challenges in terms of widening the socioeconomic gap in four key domains. These are (1) material resources (low SES students least likely to have a desk or a quiet place to study or books to help with homework); (2) digital resources (lower SES households more likely to share one computer, whereas higher SES students have multiple devices to choose from); (3) skills and dispositional characteristics (disadvantaged young people are less motivated to learn and have lower levels of resilience and perseverance); and (4) parental support (lower SES parents least likely to have the capacity to support their children’s learning). This was argued to be particularly challenging for the four in ten students from lower SES families whose parents had not completed secondary school and were therefore less likely to have access to parental support (CIRES, 2020). Parental support and, in particular, encouragement for autonomous learning have promoted greater effort towards numeracy homework (Feng et al., 2019).

Van Lancker and Parolin (2020) emphasise three particular effects of school closures: first, even in rich countries, they are associated with increased food insecurity; second, the most disadvantaged young people tend to fall furthest behind when schools close for extended periods (e.g. during the summer holidays); and third, even where schools continue teaching through online learning, the digital divide can further marginalise already disadvantaged students such as those from low SES backgrounds who tend to have inadequate access to technology (Drane et al., 2020).

## Absence and school belonging

A significant body of literature emphasises the importance of a caring environment at school in order to foster students’ engagement and sense of belonging, attendance and academic success (Cemalcilar, 2010; Korpershoek et al., 2020). While pointing out that there is no single agreed definition of school belonging, Allen et al., (2018, p. 2) argue that most definitions cover three aspects of the school environment: school-based relationships and experiences, student–teacher relationships and students’ feelings about school. García-Moya et al. (2019) argue that students’ perceptions of how adults at school care about their learning and about them as individuals is a key aspect of school belonging and an important predictor of other indicators of academic success. Roorda et al. (2011) argue that connectedness to teachers is especially important for students from low SES backgrounds if they are to have a strong sense of school belonging.

In their study of Latino students in the United States, Sánchez et al. (2005) found that absence from school significantly predicted lower school belonging. Similarly, in their examination of disengagement following transition from primary to secondary school in Canada, De Wit et al. (2010) showed a significant positive association between students’ perceptions of support from teachers and peers at school, and attendance at school. They argued that with the onset of adolescence, the importance of peer support increases for most young people. Students who do not develop a sense of belonging with the school (and do not embrace peers as sources of support) are therefore more likely to become disengaged from school, and more likely to have high rates of absence from school. However, while their analysis proposed a positive association between support and attendance, it did not suggest a direction of causality. Bowles and Scull (2019), on the other hand, proposed a theoretical causal association between absence (or non-attendance) and connectedness to school. Their review suggested that students who are chronic absentees are vulnerable to at-risk behaviour, as well as worse school outcomes, including a lower sense of belonging with peers and teachers.

This finding is consistent with Roorda et al. (2011) and Lareau (2011) who also drew strong links between SES, teacher relationships and school outcomes. Roorda et al. conducted a meta-analysis investigating the positive and negative affective associations between the teacher–student relationship and school engagement and achievement. They found significant positive associations between positive teacher–student relationships and engagement and achievement, and negative associations for negative teacher–student relationships and outcomes. The effect of SES in all these associations was stronger in samples with low SES students compared to high SES students. This indicates a stronger tendency for low SES students with positive teacher–student relationships to achieve greater gains in academic outcomes than their high SES peers. Similarly, in her qualitative account of nine fourth-grade students in the United States, Lareau found that SES was associated with how children interacted with teachers and in academic outcomes. Children in more affluent schools had access to more resources and individualised help from teachers to get through examinations, while children in schools in poorer neighbourhoods had fewer opportunities for individualised teacher support partly due to teacher shortages and higher turnover frequency. However, there is little evidence on whether SES moderates the relationship between absence and school belonging.

## Absence and academic achievement

Beyond the more recent COVID-19 crisis, literature shows a complex relationship between absence from school and academic outcomes. Carroll (2020) claims that the effect of absenteeism on learning outcomes, especially at the primary level, is under-researched and that existing research does not present a clear picture of outcomes. In his review of 15 studies, he found that seven show a positive association between absence and academic performance, while eight show a negative association. Among the latter, three show a larger effect for literacy than for numeracy, while five show a larger effect for numeracy than for literacy. Zubrick (2014) analysed the impact of absence using data from about 420,000 Western Australian (WA) primary and secondary school students across a 5-year period and found greater declines in numeracy than literacy achievement, and these effects were found to accumulate over time. Using the same data, Hancock et al. (2013) found that maternal education moderated the relationship between absence and both reading and numeracy achievement in primary school (Years 3 and 5), but not in secondary school (Years 7 and 9). Also utilising a subset of the same WA dataset, Hancock et al. (2017) found that parent occupation and education did not significantly interact with the relationship between absence and numeracy or reading among Years 5 and 7 students. However, they did observe a significant effect of absence on numeracy for students in high SES schools, but not for students in low SES schools. In a separate study of urban Australian Indigenous students, Baxter and Meyers (2019) found that school attendance was not significantly related to educational achievement. Similarly, Bein (2019), using US data from primary school students, found no significant relationship between student absence and academic achievement.

Goodman (2014), who examined absence from school in the context of snowfall in the United States, found that while school closures due to extreme snowfall did not impact student achievement (a finding that is perhaps of relevance in a COVID-19 pandemic situation), student absence where schools remained open did have a negative effect, particularly on maths achievement (an analogous situation in the current environment might be where schools are ‘open’ online, but where some students do not engage in online learning). The negative effects were stronger for students in low SES schools than for students in high SES schools. Finally, Gottfried (2009), also using US data, showed a positive relationship between *excused* absences and reading and maths achievement, while there was a strong negative relationship between *unexcused* absences and academic outcomes, a finding that Hancock et al. (2017) also report.

## Confounding factors

A wide range of factors has been shown to have an association with school belonging and academic achievement, including gender, SES (discussed above), time and effort spent on homework (Cooper et al., 2006), emotional and behavioural problems (Mundy et al., 2017), mental health (Fagel et al., 2014; Fergusson & Woodward, 2002), access to computers and Internet (Vassallo & Warren, 2018) and how they are used (e.g. for learning or entertainment) (Harris et al., 2017). These confounding factors are often related to each other. For example, Harris et al. (2017) found that despite near-equal computer access at home for their sample, young people living in disadvantaged neighbourhoods mainly used computers at home for entertainment and social activities rather than educational activities. Conversely, participants from high SES neighbourhoods more frequently used learning programmes.

Differences between boys and girls with respect to absence from school, school belonging and academic outcomes have also been extensively researched. Sánchez et al. (2005) found that among Latino students in the United States, rates of absence reported by boys and girls were similar, but that girls reported a higher sense of school belonging and greater academic achievement. However, the relationship between absence and school belonging was stronger for boys than for girls. In their study of Australian students on the other hand, Hancock et al. (2018) found that both rates of absence and effects of absence on academic achievement were similar for boys and girls.

Recently published expert reviews speculate that school closures will impact the education of low SES students particularly hard (Centre for International Research on Education Systems, 2020; Rapid Research Information Forum, 2020; Van Lancker & Parolin, 2020). However, debate on the relationship between absence, belonging and achievement is not settled. Most existing research posits a relationship between school belonging and absence where the former is seen as a precursor of the latter. Bowles and Scull (2018) theorise that physical absence from school is a precursor of a lower sense of school belonging, but do not empirically test that theory. While Carroll (2020) argues that evidence on the relationship between absence and academic outcomes is unclear, a corpus of Australian and US research suggests a stronger negative association between absence and numeracy (or maths) than between absence and reading (Goodman, 2014; Hancock et al., 2013, 2017; Zubrick, 2014). There is little agreement on the role of SES as moderating the relationship between absence from school and academic achievement, with the strongest reported effects relating to school-level SES (Goodman, 2014; Hancock et al., 2013, 2017), and little effect found for family-level SES (Hancock et al., 2017). Existing evidence does not suggest that gender significantly influences the relationship between SES, absence and achievement.

## Hypotheses

Building on theory and research findings discussed above, we propose:

### Hypothesis 1

Absence is negatively associated with school belonging and numeracy, but not reading.

### Hypothesis 2

Young people’s SES does not moderate the relationship between absence and (a) school belonging, (b) numeracy or (c) reading.

## Data

The Longitudinal Study of Australian Children (LSAC) began in May 2004, and to date, eight waves of data have been collected from two representative cohorts of randomly sampled children: a birth (B) cohort of infants born in 2003 (*N* = 5107) and a kindergarten (K) cohort of children born in 1999 (*N* = 4983). Detailed interviews relating to the study of children’s development and their social, economic and environmental contexts were conducted every two years with parents, and children themselves as they got older. The data utilised in this study were collected in 2012 and 2014 (K cohort) and 2016 and 2018 (B cohort) from the two cohorts, when adolescents were 12–13 and 14–15 years of age (henceforth ages 12 and 14 for convenience). The combined sample size at age 12 was 7337 and at age 14 was 6664.

## Measures

For the purposes of this article, *Absence* is defined as the number of times study children had been absent from school in the last 6 months. Absence was measured from responses to a single question asked directly of study children: “How many times did the following things happen to you in the last 6 months?” K Cohort respondents at age 12 responded to the statement “I was absent from school”; B Cohort respondents at age 12 and 14, and K Cohort respondents at age 14 were asked to respond to two statements: “I was absent from school *without* parental permission” and “I was absent from school *with* parental permission”. In all cases, responses were grouped into the following categories: (1) never, (2) 1–2 times, (3) 3–6 times, (4) 7–9 times and (5) 10 or more times. About 2% of B Cohort respondents reported being absent without permission at age 12, as did about 5% of B and K cohort respondents at age 14, compared with 80% or more who reported being absent with permission at both ages. To ensure fullest use of observations of both B and K cohorts, responses on absence with and without parental permission were aggregated into a continuous numerical scale which was used in the regression analysis. Sensitivity tests showed that results were broadly similar if a categorical indicator of absence was used instead (see Online Appendix Tables). Although other items in the LSAC tapping absence were asked, for example, skipping classes or being late, absence in the current study was conceptualised as non-attendance at school.

*School belonging* was conceptualised from an emotional perspective as the extent to which students have formed social bonds and feel personally accepted, included and supported at school (You et al., 2011). School belonging was measured using the Psychological Sense of School Membership (PSSM) scale (Goodenow, 1993). The PSSM measures the extent to which students feel a sense of school belonging and being personally accepted and supported as a function of school-based social bonds (Evans-Whipp & Gasser, 2019). In the LSAC, the PSSM scale is derived from responses ranging from 1 (Not at all true) to 5 (Completely true) to 12 positive and negative statements, such as “People here notice when I'm good at something”, “The teachers here respect me” and “Sometimes I don't feel as if I belong here”. In the derived scale, with values ranging from 12 to 60, negative items were reversed so that a higher score indicated a higher level of school belonging. The scale showed good reliability in the LSAC data (α = 0.85 for 12–13-year-olds; 0.87 for 14–15-year-olds).

*Academic achievement* was assessed using literacy and numeracy scores obtained from the National Assessment Program–Literacy and Numeracy (NAPLAN). NAPLAN is a standardised program that has been administered annually since 2008 as an Australian Government initiative to monitor and assess students’ academic achievements and evaluate the effectiveness of the education system (ACARA, 2019). NAPLAN scores range from about zero to 1000 (ACARA, 2016) and are linked to individual observations in the LSAC. Average NAPLAN numeracy scores among the LSAC sample at age 12 (in Year 7) were 563. This compared with 506 at age 10 (Year 5) and 608 at age 14 (Year 9). Therefore, over two years, average NAPLAN numeracy scores in the sample increased by about 29 points per year between ages 10 and 12, and by about 23 points per year between ages 12 and 14 years. Average NAPLAN reading scores increased by 45 points between ages 10 and 12 (from 514 to 559), a yearly increase of 23 points, and by 39 points (to 598) between ages 12 and 14, an annual increase of about 20 points. Therefore, differences in predicted numeracy or reading scores of 20–30 points in the analysis that follows potentially represent a difference of up to a year’s education.

### Socioeconomic status (SES)

The LSAC provides a composite indicator of family SES (called Socioeconomic Position in the LSAC data dictionary) that takes into account annual family income, years of formal education and employment status. SES scores are standardised (*Mean* = 0, *SD* = 1) with lower scores representing greater socioeconomic disadvantage.

*Control variables* used in this analysis included the following: (a) gender, (b) amount of time the young person dedicated to homework in the previous week (less than 1 h, 1–3 h, 3–5 h, 5–10 h, more than 10 h), (c) use of a computer to do homework (0 = once a month or less, 1 = more than once a month) and (d) school belonging score at age 12 and (e) NAPLAN scores at ages 10 and 12, to control for young people’s historical school belonging and learning. These variables were considered as potential confounders, given their links to school belonging and academic outcomes.

## Analytical approach

Descriptive information for all variables was summarised as means and standard deviations. Unadjusted bivariate relationships between reading and numeracy scores and school belonging scores on the one hand, and independent variables on the other, were calculated. After adjusting for control variables, a generalised linear model (GLM) was used to ascertain which factors were jointly predictive of school belonging, numeracy and reading scores. A GLM model framework was ideal for this analysis as it can deal with the problems of heteroscedasticity and skewness, which are common with education score data, by using a link function to produce a linear relationship with the predictor variables (Ho & Yu, 2015; Manning, 1998). The six models of belonging, numeracy and reading scores at ages 12 and 14 were each fitted using three families (Gaussian, Poisson and Gamma) and three link functions (identity, log and square root). The models were tested jointly using the Akaike information criterion (AIC) and the Bayesian information criteria (BIC), with lower values indicating a better fitting model. Overall, choice of family and link function had little significant influence on findings, and the Gamma family with identity link function was used in all analyses reported in this paper.

Both main effects and interaction terms of the independent variables were considered. The sign of the estimated regression coefficients for the independent variables, presented as both incremental and marginal effects, indicated whether the variable had a positive or negative relationship with the scores (Hardin & Hilbe, 2001; Stroup, 2012). Sample weights, calculated to reweight for attrition over waves, were applied to all analyses. Listwise deletion was used throughout. The GLM models' sample sizes ranged from 5939 to 6787 at age 12, and 5103 to 5756 at age 14.

**Fig. 1:**
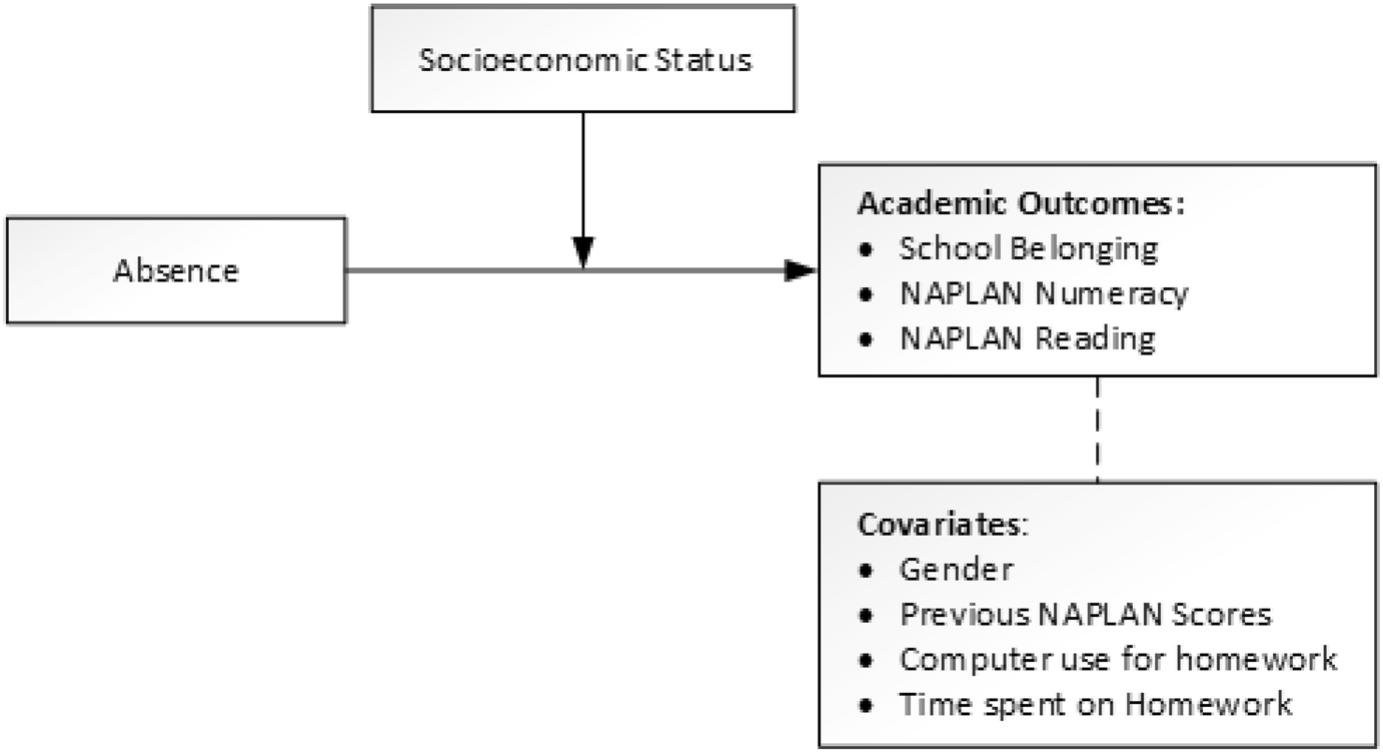
Graphical representation of the relationship between absence and academic outcomes as a function of SES

Figure 1 illustrates the relationships between absence and outcomes, with SES presented as a moderator. Covariates are also shown.

## Results

Table 1 shows means, standard deviations and correlations among variables used in this analysis, for young people aged 12 and 14. Correlations between numeracy and reading scores at Years 5 and 7 were positive and notably strong at age 12 (Year 5, *r* = .661; Year 7, *r* = .688), as were correlations of numeracy and reading scores at Years 7 and 9 among 14-year-olds (*r* = .666). Also notable is the positive and moderate relationship between SES and numeracy/reading at Years 7 and 9 (SES and numeracy: Age 12, *r* = .342; Age 14, *r* = .337. SES and reading: Age 12, *r* = .365; Age 14, *r* = .342). The association between absence and numeracy/reading at Years 7 and 9 was weak (Absence and numeracy: Age 12, *r* =  − .083; Age 14, *r* =  − .029. Absence and reading: Age 12, *r* =  − .093; Age 14, *r* =  − .044), while the association between absence and school belonging was also negative, but notably stronger (Age 12, *r* =  − .138; Age 14, *r* =  − .211.

**Table 1:** Means, standard deviations (SD) and correlations among variables

	Means	SD	1	2	3	4	5	6	7	8	9
Age 12											
1. Numeracy (Year 7)	555.91	72.84	–								
2. Reading (Year 7)	553.64	70.84	.688**	–							
3. School belonging (PSSM)	48.22	7.64	.172**	.162**	–						
4. Absence	3.21	2.97	− .083**	− .029*	− .138**	–					
5. SES	− 0.17	1.03	.342**	.365**	.140**	− .080**	–				
6. Hours spent on HW	1.91	1.00	.284**	.242**	.141**	− .095**	.221**	–			
7. Use computer for HW	0.84	0.37	.223**	.211**	.190**	− .066**	.188**		–		
8. Numeracy (Year 5)	499.84	73.02	.812**	.621**	.161**	− .066**	.320**	.208**		–	
9. Reading (Year 5)	508.55	81.22	.652**	.783**	.145**	− .033*	.339**	.204**	.235**	.661**	–
10. Female (1)	0.49	0.50	− .074**	.082**	.035*	.001	− .004	.111**	.075**	− .078**	.085**
Age 14											
1. Numeracy (Year 9)	604.74	70.68	–								
2. Reading (Year 9)	595.58	67.66	.666**	–							
3. School belonging (PSSM)	48.03	7.91	.158**	.136**	–						
4. Absence	3.67	3.25	− .093**	− .044*	− .211**	–					
5. SES	− 0.19	1.03	.337**	.342**	.137**	− .088**	–				
6. Hours spent on HW	1.98	1.10	.332**	.269**	.153**	− .120**	.212**	–			
7. Use computer for HW	0.87	0.34	.231**	.234**	.180**	− .157**	.226**	.295**	–		
8. Numeracy (Year 7)	556.82	73.06	.869**	.652**	.154**	− .075**	.333**	.233**	.297**	–	
9. Reading (Year 7)	554.64	70.44	.643**	.785**	.133**	− .053*	.354**	.276**	.225**	.681**	–
10. Female (1)	0.49	0.50	− .088**	.084**	− .082**	.016	.004	.101**	.087**	− .092**	.067**

*HW* homework. Use computer to do homework (1 = at least sometimes), *PSSM* Psychological Sense of School Membership

**p < .001, *p < .05

Table 2 shows average school belonging and NAPLAN numeracy and reading scores, by the number of times the young person reported they were absent from school for any reason. School belonging scores show a clear gradient at both ages 12 and 14, with scores decreasing monotonically and significantly as times absent increases. The Table also shows a clear gradient for numeracy, with average NAPLAN scores significantly lower among those who reported being absent even once or twice compared with those who reported no absences. Among young people who reported being absent 10 or more times, average numeracy scores, in particular, were significantly lower than was the case among students reporting no absences, with a difference of 25 points at age 12, and a difference of 28 points at age 14. On the other hand, the gradient for reading was less steep, and differences were only significant between 14-year-olds in the lowest and highest categories of absence.

**Table 2:** Average school belonging, numeracy and reading scores, by times absent last 6 months

	Age 12				Age 14			
	Per cent obs (N)	School belonging (SE)	Numeracy (SE)	Reading (SE)	Per cent obs (N)	School belonging (SE)	Numeracy (SE)	Reading (SE)
Times absent (last 6 months)								
None	12.14 (1199)	48.89 (0.23)	568.05 (2.92)	553.80 (2.55)	12.06 (762)	49.21 (0.32)	615.78 (3.57)	599.62 (3.04)
1–2	39.48 (2868)	49.10 (0.16)	557.07** (1.57)	555.74 (1.55)	39.03 (2,478)	49.18 (0.16)	609.36 (1.77)	598.01 (1.66)
3–6	31.58 (1957)	47.76*** (0.20)	553.51*** (1.81)	554.15 (1.83)	31.5 (1,982)	47.79*** (0.20)	603.12** (1.77)	596.18 (1.71)
7–9	8.19 (547)	46.09*** (0.39)	547.41*** (3.98)	552.66 (3.74)	8.27 (514)	46.94*** (0.36)	600.80** (3.85)	596.47 (3.78)
10–20	8.62 (455)	46.00*** (0.43)	543.11*** (4.11)	545.72 (4.06)	9.14 (541)	43.62*** (0.47)	587.69*** (4.11)	586.20** (3.98)

Standard errors in parentheses

***p < .001, **p < .01, *p < .05—denotes significance of difference from ‘Times absent = None’

Table 3 shows mean values of number of times absent, school belonging and NAPLAN scores, and proportions of young people who did less than 1 h or more than 5 h of homework per week, and who never used a computer to do their homework, by quintiles of SES. The Table shows that at both ages 12 and 14, young people in lower SES quintiles were on average absent more often than young people in the highest SES quintile (these differences are statistically significant). Young people in the lowest quintile were considerably more likely than young people in the highest quintile to spend less than an hour a week on homework and considerably less likely to spend five or more hours per week on homework. Almost a fifth of young people in the lowest quintile reported never using a computer to do their homework, compared with only 2% in the highest quintile. School belonging scores are shown to increase significantly with SES, as are NAPLAN numeracy and reading scores. Differences in numeracy and reading scores between young people in the lowest and highest quintiles are large and significant—74 points at age 12 and 69 points at age 14 for numeracy, and 75 points and 67 points at ages 12 and 14, respectively, for reading, representing the equivalent of about two years’ education.

**Table 3:** Absence, homework, internet access, school belonging and NAPLAN scores, by family SES

Quintile of socioeconomic status	Age 12					Age 14				
	Lowest	2nd	3rd	4th	Highest	Lowest	2nd	3rd	4th	Highest
Average times absent (SE)	3.49*** (0.10)	3.46*** (0.09)	2.98 (0.09)	3.10** (0.08)	2.80 (0.07)	4.18*** (0.12)	3.69** (0.10)	3.54 (0.10)	3.42 (0.09)	3.27 (0.09)
Does less than 1 h homework per week (%) (SE)	55.00*** (0.02)	49.00*** (0.01)	39.00*** (0.01)	35.00*** (0.01)	25.00 (0.01)	56.00*** (0.02)	50.00*** (0.02)	41.00*** (0.02)	36.00*** (0.01)	26.00 (0.01)
Does more than 5 h homework per week (%) (SE)	6.00*** (0.01)	7.00*** (0.01)	10.00*** (0.01)	10.00*** (0.01)	15.00 (0.01)	7.00*** (0.01)	8.00*** (0.01)	13.00*** (0.01)	17.00** (0.01)	23.00 (0.01)
Never used computer for homework (%) (SE)	18.00*** (0.01)	10.00*** (0.01)	9.00*** (0.01)	6.00*** (0.01)	2.00 (0.00)	17.00*** (0.01)	8.00*** (0.01)	6.00*** (0.01)	3.00 (0.01)	2.00 (0.00)
Average school belonging (PSSM) score (SE)	46.63*** (0.24)	47.93*** (0.22)	48.57*** (0.23)	49.17* (0.22)	49.81 (0.20)	46.32*** (0.27)	47.93*** (0.24)	48.62** (0.25)	48.82* (0.24)	49.54 (0.22)
Average NAPLAN numeracy score (SE)	526.49*** (2.28)	540.19*** (2.00)	558.92*** (2.19)	572.35*** (2.14)	600.90 (2.11)	578.83*** (2.37)	589.49*** (2.16)	603.44*** (2.21)	621.42*** (2.28)	647.85 (2.32)
Average NAPLAN reading score (SE)	523.25*** (2.14)	538.77*** (2.06)	555.85*** (1.97)	571.50*** (2.05)	598.66 (1.92)	570.06*** (2.31)	580.90*** (2.16)	596.01*** (2.04)	610.84*** (2.01)	637.50 (1.91)

Standard errors in parentheses

***p < .001, **p < .01, *p < .05—denotes significance of difference from highest quartile of family SES

While the unadjusted means in Table 2 suggest a significant association between times absent and both academic achievement and school belonging, the adjusted GLM estimates in Table 4 show a more nuanced picture. Note that the Pseudo-R^2^ for the NAPLAN numeracy and reading model results shown here are in the range of 0.62 to 0.76, suggesting that these models explain the majority of the variance of NAPLAN scores. Pseudo-R^2^ statistics for the school belonging model results are notably lower (0.07 and 0.30 for 12- and 14-year-olds, respectively). Table 4 shows that there was a significant negative relationship between absence and school belonging at both ages 12 and 14. On the other hand, the relationship between SES and all dependent variables was mostly positive. Hours spent doing homework was also positively and significantly associated with the dependent variables, as was using a computer at least sometimes to do homework.

**Table 4:** Estimates of association between predictor variables, school belonging, numeracy and reading scores

	Age 12			Age 14		
	Belonging	Numeracy	Reading	Belonging	Numeracy	Reading
Female	0.375* (0.179)	− 2.411* (1.082)	1.115 (1.118)	− 1.616*** (0.175)	− 3.420*** (0.968)	4.736*** (1.132)
SES (SES)						
2nd quintile	0.945* (0.391)	8.338*** (2.247)	2.65 (2.399)	1.143** (0.400)	− 2.31 (2.109)	4.604^†^ (2.527)
3rd quintile	1.947*** (0.397)	9.088*** (2.308)	8.261*** (2.478)	1.853*** (0.410)	− 1.24 (2.145)	2.111 (2.574)
4th quintile	2.249*** (0.419)	10.87*** (2.452)	11.91*** (2.635)	1.220** (0.425)	2.532 (2.227)	10.37*** (2.701)
5th quintile (Highest)	2.334*** (0.429)	20.72*** (2.572)	23.49*** (2.745)	1.424** (0.436)	6.445** (2.337)	15.98*** (2.848)
Times absent	− 0.202*** (0.053)	0.0604 (0.342)	0.246 (0.366)	− 0.299*** (0.045)	− 1.453*** (0.275)	0.103 (0.334)
SEP Q2*times absent	− 0.00562 (0.082)	− 1.141* (0.497)	0.139 (0.532)	− 0.0524 (0.074)	0.923* (0.437)	− 0.715 (0.514)
SEP Q3*times absent	− 0.224* (0.087)	− 0.526 (0.533)	0.011 (0.574)	− 0.206** (0.077)	0.622 (0.442)	0.578 (0.527)
SEP Q4*times absent	− 0.169 + (0.092)	− 0.363 (0.571)	0.249 (0.617)	− 0.115 (0.082)	0.830 + (0.465)	− 0.694 (0.573)
SEP Q5*times absent	− 0.107 (0.101)	− 1.376* (0.627)	− 0.233 (0.682)	− 0.0849 (0.086)	1.439** (0.497)	0.177 (0.603)
1–3 h homework	1.407*** (0.215)	6.670*** (1.243)	3.518** (1.337)	0.652** (0.224)	4.872*** (1.157)	4.192** (1.405)
3–5 h homework	1.628*** (0.293)	8.486*** (1.724)	6.782*** (1.840)	0.179 (0.284)	9.186*** (1.468)	5.524** (1.779)
5–10 h homework	1.459*** (0.370)	15.08*** (2.169)	8.159*** (2.325)	1.052** (0.339)	15.02*** (1.802)	6.970** (2.150)
10+ hours homework	0.748 (0.776)	19.30*** (4.627)	13.83** (4.902)	1.962*** (0.584)	16.48*** (3.116)	8.118* (3.656)
Uses computer to do homework at least sometimes	2.717*** (0.255)	9.454*** (1.527)	9.522*** (1.638)	1.614*** (0.285)	4.999** (1.607)	13.80*** (1.902)
NAPLAN—Year 5 numeracy		0.755*** (0.008)				
NAPLAN—Year 5 reading			0.608*** (0.007)			
12/13—PSSM scale				0.480*** (0.011)		
NAPLAN—Year 7 numeracy					0.787*** (0.007)	
NAPLAN—Year 7 reading						0.707*** (0.009)
Constant	44.57*** (0.324)	158.9*** (4.066)	223.7*** (3.673)	24.29*** (0.601)	159.1*** (4.153)	178.9*** (4.943)
Observations	6787	5939	5997	5756	5103	5172
Pseudo-R-squared	0.067	0.671	0.617	0.302	0.761	0.630

Standard errors in parentheses

***p < .001, **p < .01, *p < .05, ^†^*p* < 0.10

Table 4 also shows a positive and significant relationship between SES quintile membership and the three dependent variables at both ages 12 and 14. While most interaction effects of SES quintiles by absence were not significant, there was one important exception: the effect of the 5th SES quintile by absence on numeracy was significant at both ages 12 and 14.

**Fig. 2:**
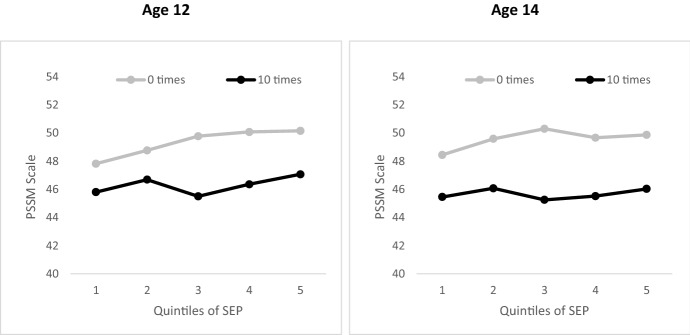
Predicted school belonging scores for young people with 0 and 10 absences from school in the previous 6 months, by quintiles of SES. Number of absence observations: Age 12, 0 times (1140) and 10 times (428); Age 14, 0 times (708) and 10 times (396)

**Fig. 3:**
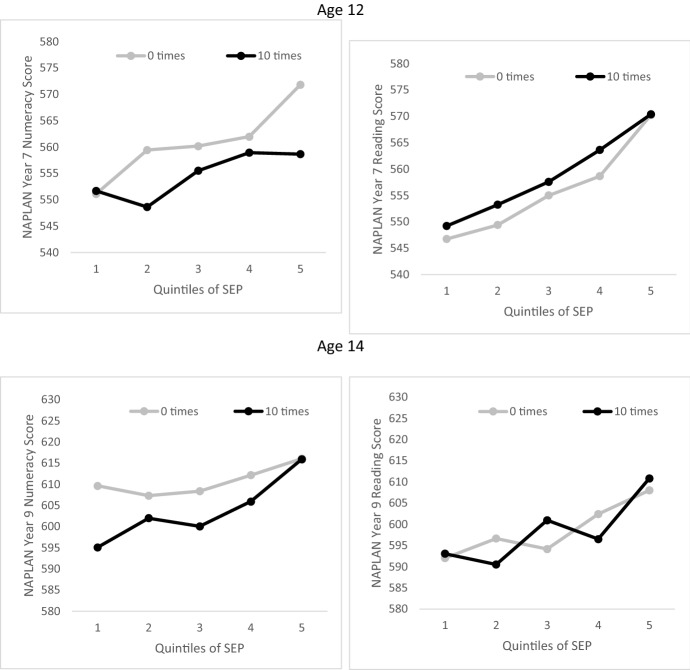
Predicted NAPLAN numeracy and reading scores for young people with 0 and 10 absences from school in the previous 6 months, by quintiles of SES. Number of absence observations: Age 12 numeracy, 0 times (1012) and 10 times (335); Age 12 reading, 0 times (1015) and 10 times (338); Age 14 numeracy, 0 times (640) and 10 times (293); Age 14 reading, 0 times (640) and 10 times (313)

Figures 2 and 3 show predicted belonging, numeracy and reading scores by quintiles of SES, where young people reported no absences, and 10 absences (numbers of observations with 0 and 10 absences are listed in notes to the Figures). Predicted scores were derived from the model presented in Table 4 using Stata *margins* command, which multiplies proportional shares by regression coefficients and calculates standard errors for resulting estimates. Figure 2 shows that the predicted school belonging scores were significantly higher across the distribution of SES at both ages 12 and 14 for young people with no absences (lighter lines) compared with young people with 10 absences (darker lines). The gap in predicted school belonging scores was largest for young people in the middle and highest SES quintiles. At age 12, predicted school belonging scores for young people with no absences were significantly higher in the top than in the bottom SES quintile (50.2 vs 47.8, *p* < .05 with Bonferroni correction). On the other hand, the difference in predicted belonging scores between young people with 10 absences in the top and bottom SES quintiles was not significant. A similar pattern was evident at age 14, with predicted belonging scores significantly higher in the top than in the bottom SES quintile for young people with no absences (49.4 vs 48.4, *p* = .004 with Bonferroni correction), but not for young people with 10 absences.

Figure 3 shows predicted NAPLAN scores for young people with no absences from school (lighter lines) and young people with 10 absences (darker lines) in the 6 months before the interview. In the reading panels for ages 12 and 14, there is a clear SES gradient for predicted NAPLAN scores at both no absences and 10 absences. However, at all points on the SES distribution, differences between young people with no absences and 10 absences were not significant. The numeracy panels on the other hand show that at age 12, predicted NAPLAN numeracy scores for young people in the lowest SES quintile are roughly equal for those reporting no absences and 10 absences. But the difference in predicted NAPLAN numeracy scores between young people with no absences and those with 10 absences in the top SES quintile was significant (571.8 vs 558.7, *p* = .048 with Bonferroni correction). At age 14, predicted NAPLAN numeracy scores for young people in the top SES quintile were almost the same whether they reported no absences or 10 absences, while predicted NAPLAN numeracy scores for young people in the bottom SES quintile with no absences were significantly higher than predicted scores for young people with 10 absences (609.6 vs 595.1, *p* < .001 with Bonferroni correction).

To identify any differences by sex, additional analysis was conducted separately for boys and girls (see the Online Appendix). Overall, results were similar for boys and girls, with the exception of some differences with respect to school belonging at age 12; among young people in the bottom SES quintile, absence was negatively and significantly associated with school belonging for girls, but not for boys; among young people in the top quintile at age 12, absence had a big effect on girls’ but not boys’ school belonging. Absence was also strongly associated with numeracy (but not reading) among top quintile girls, but not among top quintile boys. These issues may be important for future research.

The Online Appendix Tables also show results from robustness checks, where SES is included in the regression model as a continuous variable, and absence as a categorical variable. Effects are broadly the same as those presented in Figs. 2 and 3, except that in the model with continuous SES and categorical absence variables, the difference in predicted numeracy scores between young people at age 12 in the top SES quintile with no absences and 10 absences is not significant (in Fig. 3 and in the discussion above, the difference is reported as significant). On the other hand, the robustness checks and the gender analysis confirm that among both boys and girls in the bottom SES quintile, absence is negatively and significantly associated with numeracy at age 14.

## Discussion

The current investigation was conducted in the context of the COVID-19 pandemic in which physical school attendance was disrupted and partially replaced by online learning at home. The objective was to analyse some potential consequences of absence from school by examining the relationship between absence and young people’s sense of school belonging and academic outcomes, while taking into account the influence of SES. We note that the effects of absence from school where the school remains open may differ from effects in the context of school closures (Goodman, 2014). However, absence for any reason (especially prolonged absence) is of relevance in the current context.

Our first hypothesis was that absence would be negatively associated with school belonging and academic achievement in numeracy, but not reading. In accordance with the theory proposed by Bowles and Scull (2018), results indicated a negative association between absence and belonging for both 12 = and 14-year-old adolescents. For both groups, being absent 10 times in a 6-month period points to lower belonging. In terms of absence and academic outcomes, similar to Carroll (2020), results are mixed. Absence was found to have a negative association with numeracy for both 12- and 14-year-old adolescents. However, there was no association between absence and reading. This finding is partially consistent with the findings of Carroll’s review, where a link between absence and numeracy was found in a greater number of studies (five) than a link between absence and reading (three). Similarly, current findings partially concur with those of Zubrick (2014), and Hancock et al. (2013), who found increased absence to be associated with greater declines in numeracy more so than reading. Perhaps given that reading takes place in other contexts such as in different forms of media communication, email, etc., there is a tendency to read outside of school. In contrast, on the one hand, there may be fewer opportunities to engage in numeracy-based activities that utilise the maths skills expected of 12- and 14-year-olds outside of the school context and this may have potential consequent effects on the numeracy performance of poor attendees (Carroll, 2020). On the other hand, where numeracy-based opportunities do exist, these may not be explicitly recognised as such and, therefore, less focus is applied to purposefully draw upon these opportunities as numeracy-enhancing activities. Activities, for example, such as constructing things that involve measurements, angles and the like; playing or observing sport and calculating scores or distance to goal posts; and strategic gaming where one would calculate which, and how many, tokens are needed to win the game.

The negative association between absence and numeracy for 14-year-olds may also be due to heavy curricular demand and student anxiety about performing well in maths (Australian Council for Educational Research, 2018), which may be exacerbated by frequent absences from school. In addition to outcome analyses, it would be worthwhile obtaining young people’s views on curricular demand and anxiety related to numeracy to allow for more substantiated conclusions.

We expected to find that SES would not significantly impact the association between absence and sense of school belonging or academic outcomes (H2). Our findings indicate that at both ages 12 and 14, absence had the strongest negative effect on school belonging among young people in the middle SES quintiles, with the smallest effects evident among young people in the bottom SES quintiles. School belonging is recognised as helping adolescents cope with the daily challenges associated with school (Wang et al., 2019). Findings from this analysis suggest that the relationship between absence and school belonging is equally important for young people from all SES backgrounds.

We found partial support for the influence of SES on the relationship between absence and academic outcomes. The effect of the interaction between SES and absence on numeracy was significant at both ages 12 and 14, but not with respect to reading. At age 12, absence was associated with relatively lower predicted NAPLAN numeracy scores among young people in the highest SES quintile (but not consistently in all robustness checks), but not among young people in the lowest SES quintile. At age 14, absence was associated with lower predicted NAPLAN numeracy scores at the lowest but not the highest quintile (also shown consistently in robustness checks). This latter finding is somewhat different from that of Hancock et al. (2017) who reported that absence had a greater negative impact on numeracy outcomes for students from higher SES schools than less advantaged schools and that parent education and occupation were not significant predictors of the effect of absence on educational outcomes. This suggests the need for further research on how the relationship between absence and educational outcomes changes as young people transit through lower secondary school. This may be important for ensuring that disadvantaged students do not experience further disadvantage during periods of school closure or absence.

## Limitations and future directions

We did not investigate the reasons for absence. Frequent absences, whether in a block or periodic, may be linked to issues such as health or family reasons and not reflective of individual educational competencies. Given that some reasons for absence, such as absence without parental consent, have been shown to have serious negative impacts (Hancock et al., 2018), potential reasons for prolonged absence should be taken into account. As also noted by Hancock et al. (2018), a very small proportion of young people in the LSAC self-reported non-permitted absences in the previous six-month period.

An investigation of young people’s self-reported assessment of different aspects of their own schoolwork related experiences during school closure would be an important step towards gaining a deeper understanding of internal and external factors affecting educational progress. Given that early patterns of attendance are indicative of future patterns of attendance (Hancock et al., 2013; Zubrick, 2014), consequently affecting school belonging (Bowles & Scull, 2018), a deeper understanding of specific factors that promote regular attendance right through the school years is needed. It is conceivable that the academic outcomes of young people with existing attendance problems prior to school closure will have been more greatly impacted than the outcomes of regular attenders (Zubrick, 2014). Future research in this area may consider investigating the effects of school belonging, school liking and participation in extracurricular activities prior to school closures as protective assets against the risk of disengagement resulting from absence due to school closures.

Physical absence from school in the context of a pandemic such as COVID-19 has the potential to create disengagement and a lower sense of belonging with the school, particularly for those students who are falling behind academically. Minimising disruptions to students’ level of engagement with school matters as this helps to maintain young people’s sense of school belonging and educational progress (Allen et al., 2018). Online learning systems need to be efficient and up to date but will only be effective for students who have adequate access to Internet and computers at home. Academic development is also highly dependent on students’ engagement and sense of school belonging. School-based relationships such as those with teachers, peers and other school contacts need to be nurtured to promote a sense of continuity.

## Supplementary Information

Below is the link to the electronic supplementary material.

Supplementary file1 (DOCX 23 kb)
