# Supplementary material for: Does socioeconomic status impact the relationship between school absence and outcomes?
Source: Aust Educ Res. 2022 May 17;50(3):941–64. doi: 10.1007/s13384-022-00535-2 (PMC9112251; doi:10.1007/s13384-022-00535-2)
Supplement: Supplementary file 1 — Supplementary file1 (DOCX 23 kb) [file 13384_2022_535_MOESM1_ESM.docx]

Online Appendix Table A1

*Estimates of Association Between Predictor Variables, School Belonging, Numeracy and Reading scores (with continuous SEP)*

|  | Age 12 | | |  | Age 14 | | |
| --- | --- | --- | --- | --- | --- | --- | --- |
|  | Belonging | Numeracy | Reading |  | Belonging | Numeracy | Reading |
| Female | 0.231 | -2.478* | 1.363 |  | -1.804*** | -3.353*** | 4.784*** |
|  | (0.181) | (1.058) | (1.131) |  | (0.180) | (0.950) | (1.141) |
| Socioeconomic position (SEP) | 0.858*** | 5.348*** | 7.157*** |  | 0.504*** | 2.061** | 5.871*** |
|  | (0.126) | (0.769) | (0.814) |  | (0.134) | (0.743) | (0.899) |
| Times absent | -0.310*** | -0.615** | 0.254 |  | -0.390*** | -0.740*** | -0.0589 |
|  | (0.032) | (0.189) | (0.205) |  | (0.028) | (0.158) | (0.190) |
| SEP*times absent | -0.0723** | -0.12 | 0.0293 |  | -0.0391 | 0.319* | -0.18 |
|  | (0.028) | (0.186) | (0.200) |  | (0.025) | (0.153) | (0.184) |
| 1-3 hours homework | 1.414*** | 6.663*** | 3.574** |  | 0.665** | 5.034*** | 4.297** |
|  | (0.215) | (1.244) | (1.338) |  | (0.223) | (1.158) | (1.404) |
| 3-5 hours homework | 1.649*** | 8.591*** | 6.899*** |  | 0.163 | 9.401*** | 5.560** |
|  | (0.293) | (1.725) | (1.842) |  | (0.283) | (1.469) | (1.778) |
| 5-10 hours homework | 1.453*** | 15.10*** | 8.422*** |  | 1.007** | 15.47*** | 7.303*** |
|  | (0.370) | (2.171) | (2.328) |  | (0.338) | (1.801) | (2.145) |
| 10+ hours homework | 0.771 | 19.24*** | 14.09** |  | 1.905** | 16.74*** | 8.541* |
|  | (0.776) | (4.631) | (4.908) |  | (0.583) | (3.121) | (3.655) |
| Uses computer to do homework at least sometimes | 2.764*** | 9.506*** | 9.548*** |  | 1.623*** | 4.813** | 13.59*** |
|  | (0.255) | (1.527) | (1.639) |  | (0.285) | (1.606) | (1.898) |
| NAPLAN - Year 5 Numeracy |  | 0.757*** |  |  |  |  |  |
|  |  | (0.008) |  |  |  |  |  |
| NAPLAN - Year 5 Reading |  |  | 0.608*** |  |  |  |  |
|  |  |  | (0.007) |  |  |  |  |
| 12/13 - PSSM scale |  |  |  |  | 0.479*** |  |  |
|  |  |  |  |  | (0.011) |  |  |
| NAPLAN - Year 7 Numeracy |  |  |  |  |  | 0.789*** |  |
|  |  |  |  |  |  | (0.007) |  |
| NAPLAN - Year 7 Reading |  |  |  |  |  |  | 0.707*** |
|  |  |  |  |  |  |  | (0.009) |
| Constant | 46.03*** | 167.8*** | 233.0*** |  | 25.46*** | 158.8*** | 185.5*** |
|  | (0.264) | (4.076) | (3.672) |  | (0.569) | (4.171) | (4.988) |
| ---------------------- |  |  |  |  |  |  |  |
| Observations | 6787 | 5939 | 5997 |  | 5756 | 5103 | 5172 |
| Adjusted R-squared | 0.065 | 0.670 | 0.616 |  | 0.302 | 0.760 | 0.630 |

*Note*. Standard errors in parentheses. *** p<.001, ** p<.01, * p<.05.

Online Appendix Table A2

*Mean Differences in Predicted Belonging, Numeracy and Reading Scores Calculated Using Continuous and Categorical SES and Continuous and Categorical Absence for Age 12*

|  | Categorical SES & Continuous absence | |  | Continuous SES & Continuous Absence | |  | Continuous SES & Categorical Absence | |  | Boys | |  | Girls | |
| --- | --- | --- | --- | --- | --- | --- | --- | --- | --- | --- | --- | --- | --- | --- |
|  | Difference | *p* |  | Difference | *p* |  | Difference | *p* |  | Difference | *p* |  | Difference | *p* |
| **Belonging** |  |  |  |  |  |  |  |  |  |  |  |  |  |  |
| Lowest quintile | -2.02 | 0.00 |  | -2.24 | 0.00 |  | -1.71 | 0.00 |  | -0.87 | 0.22 |  | -3.31 | 0.00 |
| 2nd quintile | -2.08 | 0.00 |  | 2.67 | 0.00 |  | -2.04 | 0.00 |  | -1.98 | 0.02 |  | -2.17 | 0.02 |
| 3rd quintile | -4.26 | 0.00 |  | 3.06 | 0.00 |  | -2.34 | 0.00 |  | -3.58 | 0.00 |  | -5.19 | 0.00 |
| 4th quintile | -3.71 | 0.00 |  | 3.51 | 0.00 |  | -2.69 | 0.00 |  | -1.66 | 0.12 |  | -5.91 | 0.00 |
| Highest quintile | -3.09 | 0.00 |  | -4.05 | 0.00 |  | -3.11 | 0.00 |  | -3.52 | 0.00 |  | -2.19 | 0.11 |
| **Numeracy** |  |  |  |  |  |  |  |  |  |  |  |  |  |  |
| Lowest quintile | 0.60 | 0.86 |  | -4.73 | 0.05 |  | -5.04 | 0.12 |  | -1.63 | 0.75 |  | 2.25 | 0.62 |
| 2nd quintile | -10.80 | 0.00 |  | -5.44 | 0.00 |  | -5.34 | 0.03 |  | -20.45 | 0.00 |  | 0.84 | 0.87 |
| 3rd quintile | -4.66 | 0.26 |  | -6.10 | 0.00 |  | -5.62 | 0.03 |  | -12.14 | 0.03 |  | 5.27 | 0.38 |
| 4th quintile | -3.03 | 0.51 |  | -6.83 | 0.01 |  | -5.94 | 0.08 |  | -4.56 | 0.50 |  | -2.21 | 0.72 |
| Highest quintile | -13.15 | 0.01 |  | -7.74 | 0.03 |  | -6.32 | 0.21 |  | -4.10 | 0.57 |  | -24.99 | 0.00 |
| **Reading** |  |  |  |  |  |  |  |  |  |  |  |  |  |  |
| Lowest quintile | 2.46 | 0.50 |  | 2.19 | 0.40 |  | 2.83 | 0.42 |  | 2.83 | 0.61 |  | 2.10 | 0.67 |
| 2nd quintile | 3.85 | 0.32 |  | 2.36 | 0.25 |  | 3.12 | 0.25 |  | -2.94 | 0.59 |  | 12.05 | 0.03 |
| 3rd quintile | 2.57 | 0.56 |  | 2.52 | 0.21 |  | 3.39 | 0.22 |  | 1.04 | 0.87 |  | 5.20 | 0.42 |
| 4th quintile | 4.95 | 0.32 |  | 2.70 | 0.30 |  | 3.69 | 0.32 |  | -1.53 | 0.83 |  | 10.94 | 0.11 |
| Highest quintile | 0.13 | 0.98 |  | 2.93 | 0.44 |  | 4.07 | 0.46 |  | 1.07 | 0.89 |  | -0.88 | 0.92 |

Online Appendix Table A3

*Mean Differences in Predicted Belonging, Numeracy and Reading Scores Calculated Using Continuous and Categorical SES and Continuous and Categorical Absence for Age 14*

|  | Categorical SES & Continuous absence | |  | Continuous SES & Continuous Absence | |  | Continuous SES & Categorical Absence | |  | Boys | |  | Girls | |
| --- | --- | --- | --- | --- | --- | --- | --- | --- | --- | --- | --- | --- | --- | --- |
|  | Difference | *p* |  | Difference | *p* |  | Difference | *p* |  | Difference | *p* |  | Difference | *p* |
| **Belonging** |  |  |  |  |  |  |  |  |  |  |  |  |  |  |
| Lowest quintile | -2.99 | 0.00 |  | -3.44 | 0.00 |  | -3.63 | 0.00 |  | -4.04 | 0.00 |  | -2.05 | 0.00 |
| 2nd quintile | -3.51 | 0.00 |  | -3.67 | 0.00 |  | -3.84 | 0.00 |  | -1.72 | 0.04 |  | -4.78 | 0.00 |
| 3rd quintile | -5.05 | 0.00 |  | -3.89 | 0.00 |  | -4.03 | 0.00 |  | -3.98 | 0.00 |  | -6.41 | 0.00 |
| 4th quintile | -4.14 | 0.00 |  | -4.13 | 0.00 |  | -4.24 | 0.00 |  | -3.19 | 0.00 |  | -4.98 | 0.00 |
| Highest quintile | -3.84 | 0.00 |  | -4.41 | 0.00 |  | -4.48 | 0.00 |  | -2.69 | 0.01 |  | -5.10 | 0.00 |
| **Numeracy** |  |  |  |  |  |  |  |  |  |  |  |  |  |  |
| Lowest quintile | -14.53 | 0.00 |  | -11.19 | 0.00 |  | -12.13 | 0.00 |  | -10.18 | 0.01 |  | -18.04 | 0.00 |
| 2nd quintile | -5.30 | 0.12 |  | -9.31 | 0.00 |  | -10.95 | 0.00 |  | -3.16 | 0.53 |  | -7.86 | 0.09 |
| 3rd quintile | -8.31 | 0.02 |  | -7.48 | 0.00 |  | -9.80 | 0.00 |  | -8.72 | 0.06 |  | -7.19 | 0.17 |
| 4th quintile | -6.23 | 0.10 |  | -5.55 | 0.01 |  | -8.59 | 0.00 |  | -7.07 | 0.20 |  | -4.98 | 0.33 |
| Highest quintile | -0.14 | 0.97 |  | -3.24 | 0.25 |  | -7.14 | 0.07 |  | 0.20 | 0.97 |  | 0.02 | 1.00 |
| **Reading** |  |  |  |  |  |  |  |  |  |  |  |  |  |  |
| Lowest quintile | 1.03 | 0.76 |  | 1.54 | 0.54 |  | 3.53 | 0.33 |  | 5.90 | 0.26 |  | -3.01 | 0.48 |
| 2nd quintile | -6.12 | 0.12 |  | 0.49 | 0.80 |  | 0.95 | 0.73 |  | -11.27 | 0.05 |  | -1.19 | 0.82 |
| 3rd quintile | 6.81 | 0.10 |  | -0.54 | 0.77 |  | -1.56 | 0.56 |  | 5.00 | 0.37 |  | 9.31 | 0.13 |
| 4th quintile | -5.91 | 0.21 |  | -1.63 | 0.50 |  | -4.22 | 0.22 |  | -6.43 | 0.35 |  | -5.20 | 0.41 |
| Highest quintile | 2.80 | 0.58 |  | -2.93 | 0.39 |  | -7.40 | 0.13 |  | 8.70 | 0.22 |  | -5.18 | 0.47 |
